# Methionine and Arginine Supply Alters Abundance of Amino Acid, Insulin Signaling, and Glutathione Metabolism-Related Proteins in Bovine Subcutaneous Adipose Explants Challenged with *N*-Acetyl-d-sphingosine

**DOI:** 10.3390/ani11072114

**Published:** 2021-07-16

**Authors:** Yusheng Liang, Nana Ma, Danielle N. Coleman, Fang Liu, Yu Li, Hongyan Ding, Fabiana F. Cardoso, Claudia Parys, Felipe C. Cardoso, Juan J. Loor

**Affiliations:** 1 Department of Animal Sciences and Division of Nutritional Sciences, University of Illinois, Urbana, IL 61801, USA; yusheng4@illinois.edu (Y.L.); 15105165821@163.com (N.M.); dnc3@illinois.edu (D.N.C.); girlliufang@163.com (F.L.); lydhy2014@ahau.edu.cn (Y.L.); dinghy1988@163.com (H.D.); fabiana2@illinois.edu (F.F.C.); cardoso2@illinois.edu (F.C.C.); 2 College of Veterinary Medicine, Nanjing Agricultural University, Nanjing 210095, China; 3 Department of Animal Science and Veterinary Medicine, Henan Agricultural University, Zhengzhou 450002, China; 4 Department of Veterinary Medicine, College of Animal Science and Technology, Anhui Agricultural University, Hefei 230036, China; 5 Evonik Operations GmbH | Nutrition & Care, 63457 Hanau, Germany; claudia.parys@evonik.com

**Keywords:** amino acids, inflammation, dairy cow, oxidative stress

## Abstract

**Simple Summary:**

Increased circulating concentrations of ceramides (Ce) in dairy cows contribute to subcutaneous adipose tissue (SAT) lipolysis and could enhance the risk of developing metabolic disorders. Dietary supply of methionine (Met) or arginine (Arg) alters cellular metabolism in key tissues including SAT. Whether Met or Arg directly affect SAT metabolism when Ce concentrations are elevated is unknown. We propose that Met or Arg could have a beneficial effect within adipose tissue in terms of alleviating potential inflammatory and pro-oxidant effects associated with the transition into lactation.

**Abstract:**

The objective was to perform a proof-of-principle study to evaluate the effects of methionine (Met) and arginine (Arg) supply on protein abundance of amino acid, insulin signaling, and glutathione metabolism-related proteins in subcutaneous adipose tissue (SAT) explants under ceramide (Ce) challenge. SAT from four lactating Holstein cows was incubated with one of the following media: ideal profile of amino acid as the control (IPAA; Lys:Met 2.9:1, Lys:Arg 2:1), increased Met (incMet; Lys:Met 2.5:1), increased Arg (incArg; Lys:Arg 1:1), or incMet plus incArg (Lys:Met 2.5:1 Lys:Arg 1:1) with or without 100 μM exogenous cell-permeable Ce (*N*-Acetyl-d-sphingosine). Ceramide stimulation downregulated the overall abundance of phosphorylated (p) protein kinase B (AKT), p-mechanistic target of rapamycin (mTOR), and p-eukaryotic elongation factor 2 (eEF2). Without Ce stimulation, increased Met, Arg, or Met + Arg resulted in lower p-mTOR. Compared with control SAT stimulated with Ce, increased Met, Arg, or Met + Arg resulted in greater activation of mTOR (p-mTOR/total mTOR) and AKT (p-AKT/total AKT), with a more pronounced response due to Arg. The greatest protein abundance of glutathione S-transferase Mu 1 (GSTM1) was detected in response to increased Met supply during Ce stimulation. Ceramide stimulation decreased the overall protein abundance of the Na-coupled neutral amino acid transporter SLC38A1 and branched-chain alpha-ketoacid dehydrogenase kinase (BCKDK). However, compared with controls, increased Met or Arg supply attenuated the downregulation of BCKDK induced by Ce. Circulating ceramides might affect amino acid, insulin signaling, and glutathione metabolism in dairy cow adipose tissue. Further in vivo studies are needed to confirm the role of rumen-protected amino acids in regulating bovine adipose function.

## 1. Introduction

Alterations in subcutaneous adipose tissue (SAT) metabolism are among the key homeorhetic adaptations that characterize the peripartal period in dairy cows [1]. Lipolysis due to decreases in both insulin concentration and sensitivity, along with increased circulating ceramides [2], contributes to the onset of metabolic disorders [3,4,5]. Accumulation of the sphingolipid ceramide in the plasma and liver of peripartal cows was inversely related to markers of systemic insulin sensitivity [6]. A recent in vitro study with primary subcutaneous bovine adipocytes from beef animals indicated that a supraphysiological amount of C2:0-ceramide (100 μM) decreased activation of protein kinase B (AKT; phosphorylated (p)-AKT/total AKT) and 2-deoxy-d-(3H)-glucose uptake [7]. Thus, SAT is responsive to C2:0-ceramide and is a suitable model to study aspects of nutrient metabolism and insulin signaling in bovine SAT.

Besides its profound effect on insulin signaling in mammalian cells, downregulation of amino acid (AA) transporters by ceramides could lead to cell death [8]. A lower protein abundance of Na-dependent neutral amino acid transporter 2 (SLC38A2) along with decreased total intracellular AA concentrations were observed in rat L6 myotubes after C2:0-ceramide (100 μM) treatment for 2 h [9]. In mouse hepatocytes, C2:0-ceramide induced cell death partly by downregulating the branched-chain amino acid (BCAA) transporter SLC3A2 and intracellular content of BCAA [10]. Previous studies from our group revealed that, in addition to being an insulin-sensitive tissue [11], dairy cow SAT is a potentially important site of AA metabolism [12,13]. Whether ceramides exert any role in regulating AA uptake and intracellular AA metabolism in SAT, assessed through key molecular targets, is unknown.

The mechanistic target of rapamycin (mTOR), a key regulator of protein synthesis and cell growth and proliferation in mammals [14], is regulated by AA availability and plays multiple roles in adipose tissue [15]. For example, specific depletion of mTOR in adipose tissue caused insulin resistance in mice [16]. Across a number of studies, feeding rumen-protected Met (RPM) improved dry matter intake (DMI), milk yield, and milk protein yield around parturition [17,18,19,20]. Further, feeding RPM to achieve a Lys:Met ratio of 2.9:1 in the metabolizable protein reaching the small intestine alleviated systemic oxidative stress and ameliorated insulin insensitivity (assessed via glucose tolerance tests) [12,18,20]. The latter was supported, at least in part, by the upregulation of p-AKT in SAT of cows fed RPM [12]. Similarly, a reduction in oxidative stress in cows fed RPM was partly explained by the greater protein abundance of glutathione peroxidase, glutathione S-transferase Mu 1 (GSTM1), and p-mTOR [12].

Despite being a semi-essential AA for adult mammals, available data on Arg highlighted its significance in promoting milk protein synthesis [21] and alleviating inflammation in lactating Holstein cows challenged with lipopolysaccharide (LPS) [22]. Jugular Arg infusion prevented a decrease in plasma AA such as Ile, Leu, and Arg in cows challenged with LPS [22]. In vitro studies also reported that increased Arg supply (Lys:Arg ratio at 1:1) led to greater activation of mTOR (p-mTOR/total mTOR) in primary bovine mammary epithelial cells (BMEC) [23]. Although enhanced Arg supply downregulated mRNA abundance of solute carrier family 7 member 1 (SLC7A1), a major Arg transporter [23] in nonstimulated BMEC, it attenuated downregulation of SLC7A1 during a challenge with LPS [24]. Furthermore, enhanced Arg supply led to greater activation of mTOR (p-mTOR/total mTOR) in BMEC stimulated with LPS [25]. Thus, available data suggest that increased Arg supply could enhance lactation performance and help alleviate inflammation partly due to altered AA metabolism and mTOR activation. Of particular interest, a recent in vitro study observed that 200 μmol/L L-Arg, compared with 50 and 100 μmol/L, upregulated p-mTOR in ovine adipocyte precursor cells [26], which underscored that L-Arg could stimulate mTOR in ruminant SAT.

As the role of Arg in modulating mTOR in bovine SAT is largely unknown, and there is evidence to support a role for Met on SAT AA and glutathione metabolism, our hypothesis was that enhanced Met and/or Arg supply could counteract negative effects of Ce via increased AA transport and mTOR activation in bovine SAT. Thus, the main objective of this study was to investigate the effects of Met and Arg supply, alone or in combination, on the abundance of AA transporters and mTOR, insulin signaling, and glutathione metabolism-related proteins in bovine SAT explants challenged with C2:0-ceramide.

## 2. Methods

### 2.1. Cows

All procedures involving the handling and slaughter of cows were conducted under protocols approved by the University of Illinois Institutional Animal Care and Use Committee (Urbana; protocol # 19036). Four clinically-healthy multiparous lactating Holstein cows from the University of Illinois dairy herd that were due to be culled for being open were used. Average body weight, parity, days in milk, and milk yield prior to slaughter were 696 kg, 4, 248 d, and 27.0 kg/d, respectively. Cows had ad libitum access to the same diet formulated according to NRC (2001) [27] containing rumen-protected Met, rumen-protected Lys, and monensin once daily at 1400 h (Supplemental Tables S1 and S2). Cows were milked twice daily, housed in a free-stall barn containing sand bedding and had free access to water.

### 2.2. Tissue Collection, Processing, and Cell Culture

Cows were euthanized with a captive bolt at the College of Veterinary Medicine diagnostic laboratory facilities (University of Illinois, Urbana, IL, USA). Samples of SAT from the tailhead were obtained immediately post-slaughter and brought to the laboratory in warm Dulbecco’s modified Eagle’s medium and Ham’s F-12 nutrient mixture (DMEM:F-12; Sigma-Aldrich, St. Louis, MO, USA) containing 1% penicillin/streptomycin (Pen/Streptomycin; Sigma-Aldrich, St Louis, MO) within 30 min of collection. Subsequently, the tissue was trimmed into pieces using a sterile scalpel blade in a sterile Petri dish (catalog no. 101VR20, Thermo Fisher Scientific, Waltham, MA, USA), and then 200 mg tissue was incubated in duplicate in 5 mL of medium in 6-well plates. Culture media were: ideal profile of EAA as the control (IPAA; Lys:Met 2.9:1, Lys:Arg 2:1), increased Met (incMet; Lys:Met 2.5:1), increased Arg (incArg; Lys:Arg 1:1), or incMet plus incArg (incMet+Arg; Lys:Met 2.5:1 Lys:Arg 1:1) with or without 100 μM exogenous cell-permeable C2:0-ceramide (catalog no. A7191, Sigma-Aldrich, St Louis, MO, USA). The dose of C2:0-ceramide was based on a previous in vitro bovine adipose tissue study [7].

Ten EAAs (L-isomer, Sigma-Aldrich, St Louis, MO) were added into the custom high-glucose serum-free DMEM (devoid of these 10 EAAs, custom made from Gibco, Carlsbad, CA, USA) (Table 1). Briefly, the formulation of the EAAs was as follows: control medium with the ideal AA ratio (IPAA; Lys:Met 2.9:1; Lys: Arg 2:1; Thr:Phe 1.05:1; Lys:Thr 1.8:1; Lys:His 2.38:1; Lys:Val 1.23:1), incMet (Lys:Met 2.5:1), incArg (Lys:Arg 1:1), and incMet+Arg (Lys:Met 2.5:1; Lys:Arg 1:1). Media were prepared by increasing only Met, only Arg, or both while keeping other AA ratios the same as in IPAA. Incubations were carried out in a humidified incubator at 37 °C for 4 h with 5% CO_2_. After 4 h incubation, SAT explants were transferred from 6-well plates to screw-capped microcentrifuge tubes, snap-frozen in liquid nitrogen, and preserved at −80 °C until further analysis.

### 2.3. Western Blotting

Total protein was extracted using RIPA lysis and extraction buffer (catalog no. 89900, Thermo Fisher Scientific, Waltham, MA, USA) containing Halt protease and phosphatase inhibitor cocktail (100 ×, catalog no. 78442; Thermo Fisher Scientific), following the manufacturer’s instructions. Protein concentration was measured using the Pierce BCA protein assay kit (catalog no. 23227; Thermo Fisher Scientific, Waltham, MA, USA). Details of the Western blot procedure were reported previously [12]. Briefly, protein samples were denatured by heating at 95 °C for 5 min before loading 20 µL protein into each lane of a 4–20% SDS–PAGE gel (catalog no. 4561094; Bio-Rad, Hercules, CA, USA). Reactions were run for 10 min at 180 V and then for 45 to 60 min at 110 V. After activating a polyvinylidene fluoride membrane (catalog no. 1620261; Bio-Rad, Hercules, CA, USA) with methanol for 1 min, the protein sample was transferred to the membrane in a Trans-Blot SD Semi-Dry Electrophoretic Transfer Cell (catalog no. 170–3940; Bio-Rad, Hercules, CA, USA). Membranes were then blocked in 1 × Tris-buffered saline (TBST) containing 5% nonfat milk for 2 h at room temperature. The whole membrane was cut into small bands based on the molecular weight of target proteins. Membranes were then incubated in 1 × TBST containing primary antibodies to mTOR, p-mTOR (Ser2448), AKT, p-AKT (Ser473), eukaryotic elongation factor 2 (eEF2), p-eEF2 (Thr56), sodium-coupled neutral amino acid transporter 1 (SLC38A1), branched-chain α-keto acid dehydrogenase kinase (BCKDK), and GSTM1 overnight at 4 °C; catalog number and dilution ratios are included in Supplemental Table S3. The protein GSTM1 catalyzes the conjugation of electrophilic compounds with glutathione (GSH) to facilitate their degradation or excretion [28] and eEF2, a downstream target of mTOR, regulates the translation elongation process [29]. Antibodies for mTOR, p-mTOR (Ser2448), eEF2, p-eEF2 (Thr56) [30], AKT, p-AKT (Ser473) [11], GSTM1 [31], SLC38A1, and BCKDK [12] have been used previously in bovine samples. Membranes were then washed 6 times with 1× TBST and incubated with anti-rabbit horseradish peroxidase-conjugated secondary antibodies (catalog no. 7074S; dilution 1:800; Cell Signaling Technology, Danvers, MA, USA) for 1 h at room temperature. Subsequently, membranes were washed 6 times with 1 × TBST and then incubated with enhanced chemiluminescence reagent (catalog no. 170-5060; Bio-Rad, Hercules, CA, USA) for 3 min in the dark prior to image acquisition. Stripping and reprobing a Western blot were used for target proteins and β-actin, which have the same or similar molecular weight. β-actin (catalog no. 4967S; Cell Signaling Technology, Danvers, MA, USA) was used as the internal control. Images were acquired using the ChemiDOC MP Imaging System (Bio-Rad, Hercules, CA, USA). The intensities of the bands were measured with Image-Pro Plus 6.0 software (Media Cybernetics, Rockville, MD, USA). Specific target protein band density values were normalized to β-actin density values. Representative blots are included in Supplemental Figure S1.

### 2.4. Statistical Analysis

All data were analyzed as a 2 × 2 × 2 factorial arrangement of treatments using the MIXED procedure of SAS 9.4 (SAS Institute Inc., Cary, NC, USA). The 3 factors were Met, Arg, and C2:0-ceramide, each including 2 levels: basal or increased levels of Met or Arg and with or without C2:0-ceramide, leading to 8 treatments. The model contained the main effects of Met, Arg, and C2:0-ceramide, as well as the following interactions: Met × Arg × C2:0-ceramide, Met × C2:0-ceramide, Arg × C2:0-ceramide, and Met × Arg. The random effect was cow. Variables were assessed for normality of distribution using the Shapiro–Wilk test. Non-normally distributed data were log2-scale transformed to fit the normal distribution of residuals. Least squares means and standard errors were determined using the LSMEANS statement of SAS v.9.4 (SAS Institute Inc.) and were compared using Tukey’s test when significant interactions were observed. Significance was determined at *p* ≤ 0.05.

## 3. Results

**Figure 1:**
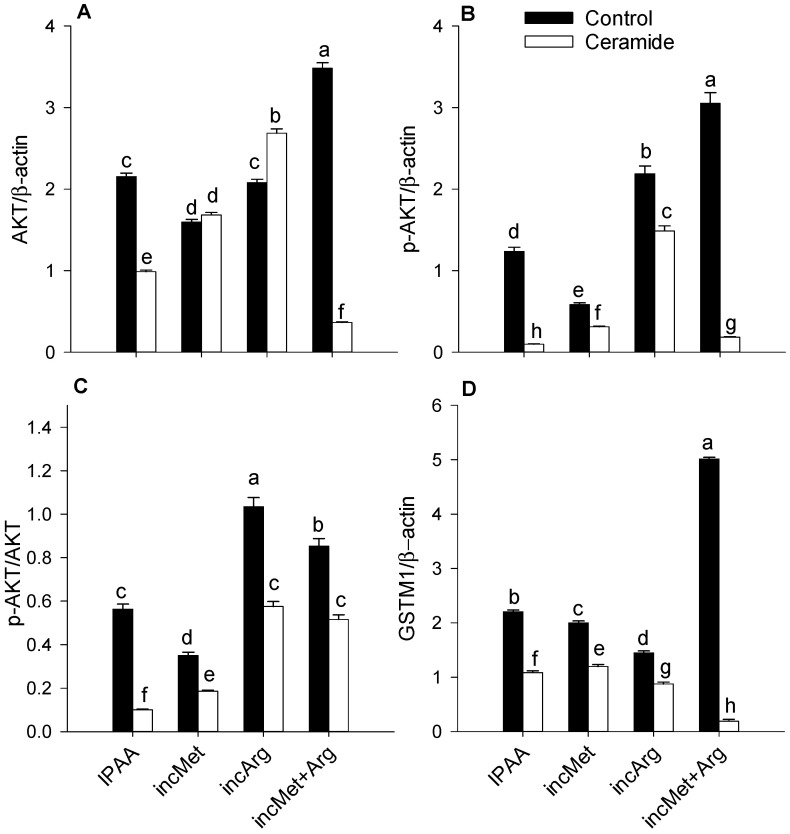
Protein abundance (relative to β-actin) of AKT (total, panel **A**), p-AKT (active, panel **B**), ratio of p-AKT/AKT (panel **C**), and GSTM1 (panel **D**) in subcutaneous adipose tissue cultured with different levels of Met or Arg and stimulated with ceramide. Control media contained an ideal AA profile (IPAA) with ratios of Lys:Met 2.9:1 and Lys:Arg 2:1. Treatment media was supplemented with greater amounts of Met or Arg to achieve ratios of Lys:Met 2.5:1 and Lys:Arg 2:1 (incMet), Lys:Met 2.9:1 and Lys:Arg 1:1 (incArg), or Lys:Met 2.5:1 and Lys:Arg 1:1 (incMet + Arg). Different letters indicate differences between treatments (Met × Arg × Ceramide *p* < 0.05). AKT = protein kinase B; GSTM1 = glutathione S-transferase Mu 1. Data are LS means, n = 4 cows per group, ±pooled SEMs.

**Figure 2:**
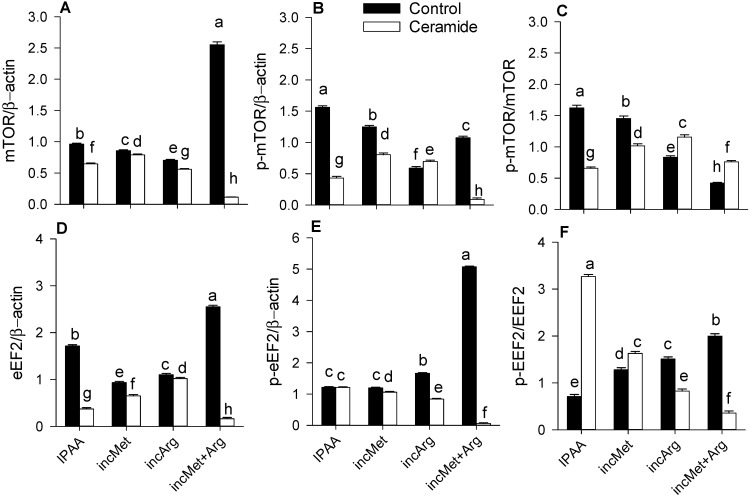
Protein abundance (relative to β-actin) of mTOR (total, panel **A**), p-mTOR (active, panel **B**), ratio of p-mTOR/mTOR (panel **C**) eEF2 (total, panel **D**), p-eEF2 (active, panel **E**), and ratio of p-eEF2/eEF2 (panel **F**) in subcutaneous adipose tissue cultured with different levels of Met or Arg and stimulated with ceramide. Control media contained an ideal AA profile (IPAA) with ratios of Lys:Met 2.9:1 and Lys:Arg 2:1. Treatment media were supplemented with greater amounts of Met or Arg to achieve ratios of Lys:Met 2.5:1 and Lys:Arg 2:1 (incMet), Lys:Met 2.9:1 and Lys:Arg 1:1 (incArg), or Lys:Met 2.5:1 and Lys:Arg 1:1 (incMet + Arg). Different lowercase letters indicate differences between treatments (Met × Arg × Ceramide *p* < 0.05). mTOR = mechanistic target of rapamycin; eEF2 = eukaryotic elongation factor 2. Data are LS means, n = 4 cows per group, ±pooled SEMs.

Ceramide stimulation downregulated overall abundance of p-AKT, p-mTOR and p-eEF2 (*p* < 0.01; *p* < 0.01; *p* < 0.01; Table 2). There was a Met × Arg × Ceramide interaction for p-AKT, p-mTOR and p-eEF2 (*p* < 0.01; *p* < 0.01; *p* < 0.01; Figure 1B and Figure 2B,E). Without ceramide challenge, enhanced supply of Met and Arg alone or in combination led to lower p-mTOR (*p* < 0.01; *p* < 0.01; *p* < 0.01; Figure 2B). However, compared with the control (IPAA) cultures challenged with ceramide, enhanced Met or Arg supply alone or in combination resulted in greater activation of AKT (p-AKT/total AKT) and mTOR (p-mTOR/total mTOR) (*p* < 0.01; *p* < 0.01; Figure 1C and Figure 2C), with a more pronounced response due to Arg (*p* < 0.01; Figure 1C and Figure 2C). In contrast, compared with IPAA challenged with ceramide, enhanced Met or Arg supply alone or in combination led to lower activation of eEF2 (p-eEF2/total eEF2) (*p* < 0.01; *p* < 0.01; *p* < 0.01; Figure 2F). Ceramide stimulation downregulated overall abundance of GSTM1 (*p* < 0.01; Table 2). A Met × Arg × Ceramide interaction was observed for GSTM1 (*p* < 0.01; Figure 1D). It is noteworthy that greater Met supply alone during ceramide challenge led to the greatest abundance of GSTM1 (*p* < 0.01; Figure 1D).

**Figure 3:**
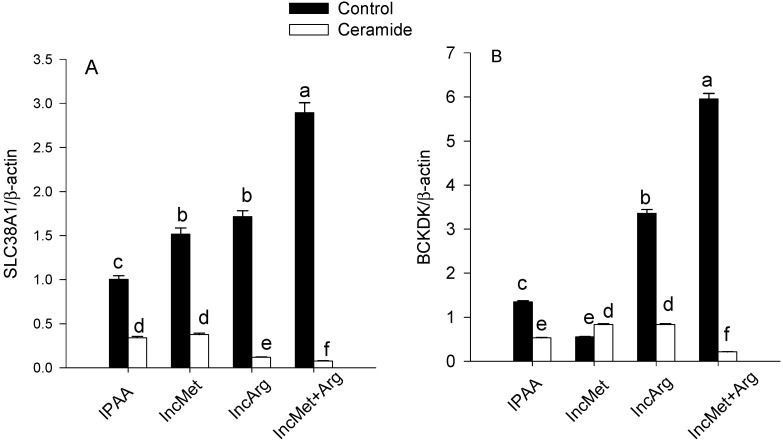
Protein abundance (relative to β-actin) of SLC38A1 (panel **A**) and BCKDK (panel **B**) in subcutaneous adipose tissue cultured with different levels of Met or Arg and stimulated with ceramide. Control media contained an ideal AA profile (IPAA) with ratios of Lys:Met 2.9:1 and Lys:Arg 2:1. Treatment media were supplemented with greater amounts of Met or Arg to achieve ratios of Lys:Met 2.5:1 and Lys:Arg 2:1 (incMet), Lys:Met 2.9:1 and Lys:Arg 1:1 (incArg), or Lys:Met 2.5:1 and Lys:Arg 1:1 (incMet + Arg). Different letters indicate differences between treatments (Met × Arg × Ceramide *p* < 0.05). SLC38A1 = solute carrier family 38 member 1; BCKDK = branched-chain α-keto acid dehydrogenase kinase. Data are LS means, n = 4 cows per group, ±pooled SEMs.

A triple interaction between Met, Arg and ceramide was observed for the protein abundance of SLC38A1 and BCKDK (*p* < 0.01; *p* < 0.01; Figure 3). Ceramide stimulation reduced overall protein abundance of SLC38A1 and BCKDK (*p* < 0.01; *p* < 0.01; Table 2). With ceramide stimulation, compared with IPAA, greater supply of Met or Arg alone attenuated the downregulation of BCKDK (*p* < 0.01; *p* < 0.01; Figure 3B).

## 4. Discussion

The data indicated that C2:0-ceramide had an inhibitory effect on SLC38A1 abundance and led to reduced activation of AKT in SAT. In contrast, enhancing the supply of Arg and Met alone contributed to increasing protein abundance of BCKDK, a rate-limiting enzyme of BCAA catabolism, and mTOR pathway activity in bovine adipose explants stimulated with C2:0-ceramide.

An observational study concluded that there is a negative association between oxidative stress and insulin sensitivity in peripartal cows [32]. Although it is unclear whether a Lys:Met ratio of 2.8:1–2.9:1 has beneficial effects in late-lactation cows, our previous studies consistently demonstrated that achieving a Lys:Met ratio of 2.8:1–2.9:1 at the intestine by feeding RPM during the peripartal period reduced oxidative stress and inflammation status [18,19,20]. A key feature of those studies was the greater and more consistent DMI and its effect on the synthesis of GSH and taurine, both of which are sulfur-containing antioxidants [17].

Specifically in SAT, compared with controls, peripartal cows fed RPM had greater protein abundance of p-AKT (a key regulator of insulin signaling) and GSTM1, suggesting that these proteins might be crucial during the peripartal period [12,33]. Dairy cows with greater oxidative stress status exhibited reduced protein abundance of GSTM1 in SAT [31,33], suggesting that GSTM1 might play a role in alleviating oxidative stress in bovine SAT.

In the current study, the lower GSTM1, coupled with lower activation of p-AKT in SAT during stimulation with C2:0-ceramide, suggested that this metabolite could impair insulin signaling and may also disrupt redox balance in SAT. Although increased Met had no overall effect on the activation of AKT, it is noteworthy that the greatest protein abundance of GSTM1 in response to enhanced Met supply was observed in SAT stimulated with ceramide suggesting that enhanced Met itself might play a positive role within SAT under physiological conditions that increased concentrations of these compounds.

In nonruminants, it is well-established that the mTOR signaling pathway is a key regulator of protein synthesis, cell growth, and proliferation [34]. Amino acids such as Gln, Arg, Met, and BCAA (particularly Leu) can directly or indirectly activate mTOR [35,36]. Despite the fact that greater Met and Arg supply alone or in combination did not reverse the reduction of SLC38A1 abundance under ceramide stimulation, compared with IPAA (control), greater Met and Arg supply alone led to greater protein abundance of BCKDK. In nonruminant cells, BCKDK is a rate-limiting enzyme regulating BCAA catabolism via inactivation and phosphorylation of the BCKD complex [37]. Thus, greater protein abundance of BCKDK contributes to decreasing intracellular BCAA catabolism. We speculate that greater abundance of BCKDK in response to increased Met or Arg alone during stimulation with ceramide partly explains the greater activation of mTOR (p-mTOR/total mTOR) and lower activation of eEF2 (p-eEF2/total eEF2). Taken together, our results suggested that increased ceramide concentrations inhibit SLC38A1 abundance in SAT, which is likely to cause an intracellular deficiency in AA supply.

Some limitations of the present study should be acknowledged. First, by design, this study narrowly focused on few components of the AA, insulin signaling, and glutathione metabolism-related pathways during C2:0-ceramide stimulation under basal conditions. Thus, the effect of insulin per se could not be clarified and will require, for example, an insulin challenge. Second, in spite of its use in similar studies [7], a supraphysiological concentration of C2:0-ceramide was used, and this compound is not found naturally in vivo. Thus, responses detected may not adequately reflect the ability of naturally occurring, longer-chain ceramides to modify insulin signaling in SAT. Lastly, adipose tissue was obtained from late-lactation cows, which cannot reflect the responsiveness to Met and Arg in SAT obtained from peripartal cows.

## 5. Conclusions

Overall, data suggested that stimulation with C2:0-ceramide could inhibit AA uptake by adipose tissue and decrease insulin signaling. Under those conditions, enhancing the supply of Arg or Met contributed to altering AA metabolism by increasing the protein abundance of BCKDK and mTOR pathway activity. Unique responses to Arg and Met supply during ceramide stimulation included greater activation of mTOR by Arg, while Met increased the antioxidant response through upregulation of GSTM1. Future in vivo work is warranted to investigate how AA modulate the effect of longer-chain ceramides on insulin signaling in dairy cows, especially during the peripartal period. The focus should be placed on both SAT and immune cell activation.

## Figures and Tables

**Table 1:** Amino acid (AA) composition of the culture media with an ideal profile of AA (IPAA) and treatment media supplemented with greater amounts of Arg (incArg), Met (incMet), or both (incMet + incArg) to alter the ratio of Lys:Met and Lys:Arg relative to IPAA.

Amino Acid	IPAA ^1^	IncMet ^2^	IncArg ^3^	IncMet + IncArg ^4^
L-Lys (µg/mL)	175	175	175	175
Lys:Met	2.9:1	2.5:1	2.9:1	2.5:1
Lys:Arg	2:1	2:1	1:1	1:1
L-Met (µg/mL)	60	70	60	70
L-Arg (µg/mL)	84	84	175	175
L-His (µg/mL)	74	74	74	74
L-Ile (µg/mL)	121	121	121	121
L-Leu (µg/mL)	206	206	206	206
L-Phe (µg/mL)	93	93	93	93
L-Thr (µg/mL)	97	97	97	97
L-Trp (µg/mL)	16	16	16	16
L-Val (µg/mL)	142	142	142	142

^1^ IPAA = ideal profile of AA, used as control medium. Ratios of essential AA are as follows: Lys:Met = 2.9, Lys:Thr = 1.8, Lys:His = 2.38, Lys:Val = 1.23, and Thr:Phe = 1.05, and were based on NRC (2001) [27] and our previous studies [24]. ^2^ Composition of AA in the medium was prepared, as described in our previous study [24]. ^3^ Level of Met was based on previous in vitro studies from our laboratory [24]. ^4^ Level of Arg was based on previous in vitro studies from our laboratory [24].

**Table 2:** Abundance of proteins (relative to β-actin) related to the mechanistic target of rapamycin (mTOR) and insulin signaling pathways in subcutaneous adipose tissue cultured with different levels of Met or Arg and challenged with ceramide ^1^.

Item ^2^	Met			Arg			Cer			*p*-Value					
	No	Yes	SEM	No	Yes	SEM	No	Yes	SEM	Met	Arg	Cer	Met × Arg	Met × Cer	Arg × Cer
Insulin signaling															
AKT/β-actin	1.86	1.36	0.02	1.55	1.63	0.02	2.24	1.13	0.02	<0.01	<0.01	<0.01	<0.01	<0.01	<0.01
p-AKT/β-actin	0.80	0.57	0.02	0.39	1.16	0.03	1.48	0.30	0.04	<0.01	<0.01	<0.01	<0.01	<0.01	0.63
p-AKT/AKT	0.43	0.41	0.01	0.25	0.72	0.02	0.65	0.27	0.02	0.12	<0.01	<0.01	<0.01	<0.01	<0.01
Glutathione metabolism															
GSTM1/β-actin	1.40	2.10	0.02	1.62	1.88	0.02	2.66	0.84	0.02	<0.01	<0.01	<0.01	<0.01	<0.01	<0.01
Amino acid metabolism															
BCKDK/β-actin	1.19	0.88	0.01	0.76	1.37	0.01	1.97	0.53	0.02	<0.01	<0.01	<0.01	<0.01	<0.01	<0.01
SLC38A1/β-actin	0.51	0.60	0.01	0.67	0.46	0.01	1.66	0.19	0.03	<0.01	<0.01	<0.01	<0.01	<0.01	<0.01
mTOR pathway															
mTOR/β-actin	0.71	0.67	0.01	0.81	0.59	0.01	1.10	0.43	0.01	<0.01	<0.01	<0.01	<0.01	<0.01	<0.01
p-mTOR/β-actin	0.82	0.81	0.01	1.01	0.61	0.01	1.12	0.51	0.01	0.41	<0.01	<0.01	0.02	<0.01	<0.01
p-mTOR/mTOR	1.01	0.83	0.01	1.12	0.75	0.02	0.95	0.88	0.01	<0.01	<0.01	<0.01	<0.01	<0.01	<0.01
eEF2/β-actin	1.05	1.08	0.01	0.92	1.21	0.01	1.58	0.55	0.01	0.25	<0.01	<0.01	<0.01	<0.01	<0.01
p-eEF2/β-actin	1.23	1.85	0.01	1.17	1.91	0.01	2.28	0.79	0.01	<0.01	<0.01	<0.01	<0.01	<0.01	<0.01
p-eEF2/eEF2	1.58	1.31	0.02	1.72	1.17	0.02	1.37	1.52	0.02	<0.01	<0.01	<0.01	<0.01	<0.01	<0.01

^1^ −Arg, −Met = cultures without increased supply of Arg or Met; + Arg, + Met = cultures with increased supply of Arg or Met; −/+ C2:0-ceramide (Cer), cultures without or with Cer stimulation. Control media contained an ideal AA profile with ratios of Lys:Met 2.9:1 and Lys:Arg 2:1. Treatment media was supplemented with greater amounts of Met or Arg to achieve ratios of Lys:Met 2.5:1 and Lys:Arg 2:1 (incMet), Lys:Met 2.9:1 and Lys:Arg 1:1 (incArg), or Lys:Met 2.5:1 and Lys:Arg 1:1 (incMetArg). Each treatment was challenged with or without 100 μM Cer. Data are LS means, n = 4 cows per group, ±pooled SEMs. Three-way interactions are depicted in Figure 1, Figure 2 and Figure 3. ^2^ AKT = protein kinase B; GSTM1 = glutathione S-transferase mu 1; BCKDK = branched-chain α-keto acid dehydrogenase kinase; SLC38A1 = solute carrier family 38 member 1; mTOR = mechanistic target of rapamycin; eEF2 = eukaryotic elongation factor 2.

## Data Availability

The data reported in this manuscript are available upon reasonable request from the corresponding author (J.J.L.).

## Supplementary Materials

The following are available online at https://www.mdpi.com/article/10.3390/ani11072114/s1. Table S1: Ingredient composition of the lactation diet fed to cows, Table S2: Chemical composition and associated standard deviations for diets fed to cows, Table S3: Catalog number and source, dilution ratios, and target protein antibodies used in the present study, Figure S1: Representative blots with band size information.

## Abbreviations

AA	Amino acid
AKT	Protein kinase B
Arg	Arginine
BCKDK	Branched-chain alpha-ketoacid dehydrogenase kinase
BCAA	Branched-chain amino acids
eEF2	Eukaryotic elongation factor 2
GSTM1	Glutathione S-transferase mu 1
incMet	Increased Met
incArg	Increased Arg
incMet + Arg	Increased Met plus increased Arg
IPAA	Ideal profile of AA
Met	Methionine
mTOR	Mechanistic target of rapamycin
NRC	National research council
RPM	Rumen-protected Met
SAT	Subcutaneous adipose tissue
SLC38A1	Na-coupled neutral amino acid transporter
