# Supplementary material for: Methionine and Arginine Supply Alters Abundance of Amino Acid, Insulin Signaling, and Glutathione Metabolism-Related Proteins in Bovine Subcutaneous Adipose Explants Challenged with N-Acetyl-d-sphingosine
Source: Animals (Basel). 2021 Jul 16;11(7):2114. doi: 10.3390/ani11072114 (PMC8300206; doi:10.3390/ani11072114)
Supplement: Supplementary file 1 [file animals-11-02114-s001.zip › animals-1273517-supplementary.pdf]

Supplemental Table S1. Ingredient composition of the lactation diet fed to cows prior to slaughter.

Ingredient	% of DM
Corn silage	40.2
Dry ground corn grain	17.3
Canola meal expelled	5.6
Alfalfa hay	18.4
Corn gluten feed pellets	7.9
Vitamin and mineral mix[1]	4.4
Blood meal[2]	0.4
Rumen-protected lysine[3]	0.4
Rumen-protected methionine[4]	0.1
Urea 281 CP	0.4
Rumen inert fat[5]	1.5
Molasses	3.4

[1]Mineral and vitamin mix was formulated to contain 12.51% Ca, 14.06% Na, 9.60% Cl, 3.18% Mg, 6.48% K, 0.19% S, 26.93 mg/kg Co, 301.01 mg/kg of Cu, 40.22 mg/kg of I, 678.25 mg/kg Fe, 1,519.35 mg/kg Mn, 8.62 mg/kg Se, 4.47 mg/kg of organic Se, 1621.05 mg/kg of Zn, 43.34 kIU/kg Vitamin A, 10.89 kIU/kg of Vitamin D_3, 466.41 IU/kg of Vitamin E, 4.23 mg/kg of biotin, 46.65 mg/kg of thiamine, and 0.35 g/kg of monensin (Rumensin, Elanco, Greenfield, IN)
[2]ProVAAl AADvantage (Perdue AgriBusiness, Salisbury, MD)
[3]Ajipro-L Generation 3 (Ajinomoto Heartland, Inc., Chicago, IL)
[4]Smartamine M (Adisseo, Alpharetta, GA).
[5]Energy Booster 100 (Milk Specialties Global, Eden Prairie, MN)

Supplemental Table S2. Chemical composition and associated standard deviations for diets fed to cows prior to slaughter.

Item	Mean	SD
DM, %	49.83	3.74
CP, % of DM	15.74	0.75
ADF, % of DM	20.21	1.92
NDF, % of DM	30.66	1.80
Lignin, % of DM	3.74	0.70
NFC, % of DM	41.81	2.22
Starch, % of DM	27.19	3.23
Crude fat, % of DM	4.30	0.32
Ash, % of DM	7.44	0.90
NE_L, Mcal/kg of DM	1.67	0.02
Ca, % of DM	0.82	0.13
P, % of DM	0.44	0.03
Mg, % of DM	0.29	0.02
K, % of DM	1.59	0.14
Na, % of DM	0.56	0.08
S, % of DM	0.25	0.01
Fe, ppm	260	81
Zn, ppm	101.86	14.44
Cu, ppm	13.57	1.72
Mn, ppm	76.14	7.08
Mo, ppm	1.03	0.36

Supplemental Table S3. Catalog number and source, dilution ratios, and target protein antibodies used in the present study.

Antibody	Catalog Number	Company	Dilution ratio	Antibody Name
mTOR	2972S	Cell Signaling Technology	1:500	Mechanistic target of rapamycin
Phospho-mTOR (Ser2448)	2971S	Cell Signaling Technology	1:250	Phosphorylated mechanistic target of rapamycin
AKT	9272S	Cell Signaling Technology	1:500	Protein kinase B
Phospho-AKT (Ser473)	9271S	Cell Signaling Technology	1:250	Phosphorylated protein kinase B
GSTM1	ARP41769_P050	Aviva Systems Biology	1:500	Glutathione S-transferase Mu 1
SLC38A1	ab60145	Abcam	1:250	Solute carrier family 38 member 1
BCKDK	ab151297	Abcam	1:500	Branched-chain α-keto acid dehydrogenase kinase
eEF2	2332s	Cell Signaling Technology	1:500	Eukaryotic elongation factor 2
Phospho-eEF2 (Thr56)	2331s	Cell Signaling Technology	1:250	Phosphorylated eukaryotic elongation factor 2

Supplemental Figure S1. Representative blots with band size information.

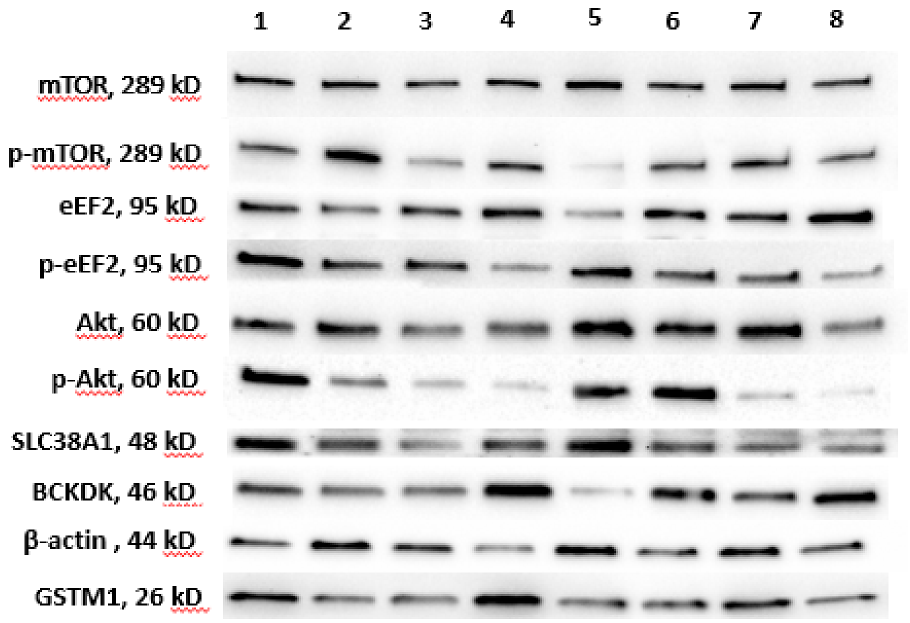

1. -Met,-Arg, -C2:0-ceramide 2. +Met,-Arg, -C2:0-ceramide 3. -Met,-Arg, +C2:0-ceramide
4. +Met,-Arg,+C2:0-ceramide 5. -Met,+Arg,-C2:0-ceramide 6.+Met,+Arg, -C2:0-ceramide
7. -Met,+Arg,+C2:0-ceramide 8.+Met,+Arg, +C2:0-ceramide
